# Dietary management of dyslipidemia in HIV-infected young adults in antiretroviral treatment

**DOI:** 10.1186/1471-2334-14-S7-P24

**Published:** 2014-10-15

**Authors:** Maria Nițescu, Adrian Streinu-Cercel, Ana Maria Tudor

**Affiliations:** 1 National Institute for Infectious Diseases "Prof. Dr. Matei Balş", Bucharest, Romania; 2 Carol Davila University of Medicine and Pharmacy, Bucharest, Romania

## Background

HIV infection is a manageable disease with antiretroviral treatment, but this comes with the cost of metabolic disturbances. The most frequent issues are dyslipidemia and lipodystrophy. The scientific papers highlight the effectiveness of weight loss and healthy lifestyle to reduce serum lipids levels. Objective: to assess the effectiveness of nutritional intervention on dyslipidemia in HIV-infected young adults.

## Methods

HIV-infected young adults on antiretroviral treatment, with a follow-up of at least 10 years in the National Institute for Infectious Diseases "Prof. Dr. Matei Balş", were evaluated (anthropometric and biochemical measurements). The dietary habits and physical activity were assessed using a questionnaire administered by an interviewer. We drew up a healthy nutrition plan based on energetic needs, to maintain or to lose weight.

## Results

We assessed 18 patients, but only 11 of them (8 males/3 females, 25 years old) gave their written consent to nutritional counseling. Overweight and obesity were detected in 54.6% of cases. With respect to dietary habits, 81% had a high intake of saturated fats, 72% had a high intake of added sugar and 100% had a low intake of fibers. The most frequent types of food eaten were fast-food, maturated cheese, sweets and soft drinks. More than 80% had sedentary behavior. The most frequent laboratory abnormality was high level of serum triglycerides 10/11 cases, 5 cases associated high levels of serum total cholesterol with 4 high low-density lipoprotein cholesterol (LDL-c) and 9 cases had low high-density lipoprotein cholesterol (HDL-c).

Only in two cases, we have completed 6 weeks of follow-up and we performed a clinical and laboratory evaluation. In the first case, the patient followed all recommendations and the value of triglycerides and cholesterol were back to normal and he lost weight (6 kg). In the second case, the patient followed almost all recommendations, except that instead of gas sodas she drank non-carbonated sweetened juices. So, in this case the triglycerides did not return to normal, but we still noticed a lower level compared to baseline.

## Conclusion

Nutritional counseling for overweight/obesity and dyslipidemia in HIV infected patients is cheaper than lipid-lowering drugs and it had no effects on antiretroviral treatment. We need a longer time of follow-up and more patients to be able to draw conclusions, to see if the duration of the effect on metabolism is durable and to find a proper dietary pattern which may prevent or undo antiretroviral side effects.

